# Efficacy of extracorporeal membrane oxygenation for acute respiratory failure with interstitial lung disease: a case control nationwide dataset study in Japan

**DOI:** 10.1186/s12931-021-01805-w

**Published:** 2021-07-24

**Authors:** Yuko Usagawa, Kosaku Komiya, Mari Yamasue, Kiyohide Fushimi, Kazufumi Hiramatsu, Jun-ichi Kadota

**Affiliations:** 1 Department of Respiratory Medicine and Infectious Diseases, Faculty of Medicine, Oita University, 1-1 Idaigaoka, Hasama-machi, Yufu, Oita 879-5593 Japan; 2 Department of Health Policy and Informatics, Tokyo Medical and Dental University Graduate School of Medicine, Tokyo, Japan; 3 Department of Medical Safety Management, Faculty of Medicine, Oita University, Oita, Japan

**Keywords:** Extracorporeal membrane oxygenation, Interstitial lung disease, Macrolides, Mortality, Respiratory failure

## Abstract

**Background:**

Whether acute respiratory failure in patients with interstitial lung disease is reversible remains uncertain. Consequently, indications for extracorporeal membrane oxygenation in these patients are still controversial, except as a bridge to lung transplantation. The objective of this study was to clarify in-hospital mortality and prognostic factors in interstitial lung disease patients undergoing extracorporeal membrane oxygenation.

**Methods:**

In this case–control study using the Japanese Diagnosis Procedure Combination database, hospitalized interstitial lung disease patients receiving invasive mechanical ventilation and extracorporeal membrane oxygenation from 2010 to 2017 were reviewed. Patients’ characteristics and treatment regimens were compared between survivors and non-survivors to identify prognostic factors. To avoid selection biases, patients treated with extracorporeal membrane oxygenation as a bridge to lung transplantation were excluded.

**Results:**

A total of 164 interstitial lung disease patients receiving extracorporeal membrane oxygenation were included. Their in-hospital mortality was 74.4% (122/164). Compared with survivors, non-survivors were older and received high-dose cyclophosphamide, protease inhibitors, and antifungal drugs more frequently, but macrolides and anti-influenza drugs less frequently. On multivariate analysis, the following factors were associated with in-hospital mortality: advanced age (odds ratio [OR] 1.043; 95% confidence interval [CI] 1.009–1.078), non-use of macrolides (OR 0.305; 95% CI 0.134–0.698), and use of antifungal drugs (OR 2.416; 95% CI 1.025–5.696).

**Conclusions:**

Approximately three-quarters of interstitial lung disease patients undergoing extracorporeal membrane oxygenation died in hospital. Moreover, advanced age, non-use of macrolides, and use of antifungal drugs were found to correlate with a poor prognosis.

## Background

Regardless of the type of interstitial lung disease (ILD), the associated acute respiratory failure (ARF) leads to a poor prognosis, especially in patients requiring invasive mechanical ventilation (IMV) [1–3]. Extracorporeal membrane oxygenation (ECMO), also known as extracorporeal life support, is a life-saving procedure established for severe respiratory failure, cardiac shock, and cardiac arrest. Veno-venous ECMO serves as an artificial lung that provides oxygenation and removes carbon dioxide by draining and reinfusing the blood through cannulas located in central veins. ECMO enables lung-protective ventilation and reduces complications such as ventilator-induced lung injury and oxygen toxicity [4]. Ever since ECMO therapy proved successful in influenza A (H1N1)-induced severe acute respiratory distress syndrome (ARDS) [5], as well as severe adult respiratory failure (CESAR trial) [6] in 2009, the use of ECMO for respiratory failure in adults has expanded rapidly.

ECMO therapy is indicated in patients with potentially reversible causes of respiratory failure or those awaiting lung transplantation [7, 8]. Due to difficulties in predicting the reversibility of respiratory failure, which arise from its heterogeneous causes, the indications for ECMO in ILD patients with ARF need to be discussed. Moreover, even in-hospital mortality and prognostic factors in ILD patients receiving ECMO have not been clarified. The aim of this study was to elucidate the mortality rate and identify prognostic factors in these patients by using nationwide data from the Japanese Diagnosis Procedure Combination (DPC) database.

## Methods

### Study design and data source

This was a retrospective, case–control study using a nationwide inpatient database of acute care hospitals in Japan (i.e., the DPC database) to assess the efficacy of ECMO therapy for ARF in ILD patients treated between 2010 and 2017. The DPC is a case-mix classification system that is linked with a lump-sum payment system for inpatient care reimbursement. The Japanese DPC database includes data on the following characteristics: age, sex, weight, admission and discharge status, main diagnosis, admission-precipitating diagnosis, resource-consuming diagnosis, comorbidities, complications, surgery, and procedures performed and medications administered during hospitalization. Diagnoses are recorded using International Classification of Diseases, 10th revision, codes by attending physicians. Surgery and procedures performed during hospitalization are recorded according to the Japanese fee schedule for reimbursement.

In April 2020, this system included 1,757 hospitals with a total of 483,180 beds, which covers nearly all acute inpatients in Japan [9]. The specificity and sensitivity of diagnoses and procedures recorded in the DPC database have already been validated by Yamana et al. [10]. Since all patient data were obtained in an anonymous manner, the requirement for individual informed consent was waived. This study was approved by the institutional review board of the General Clinical Research Center, Oita University Hospital, on May 20, 2019 (approval no. 1613).

### Patient selection and data extraction

**Fig. 1:**
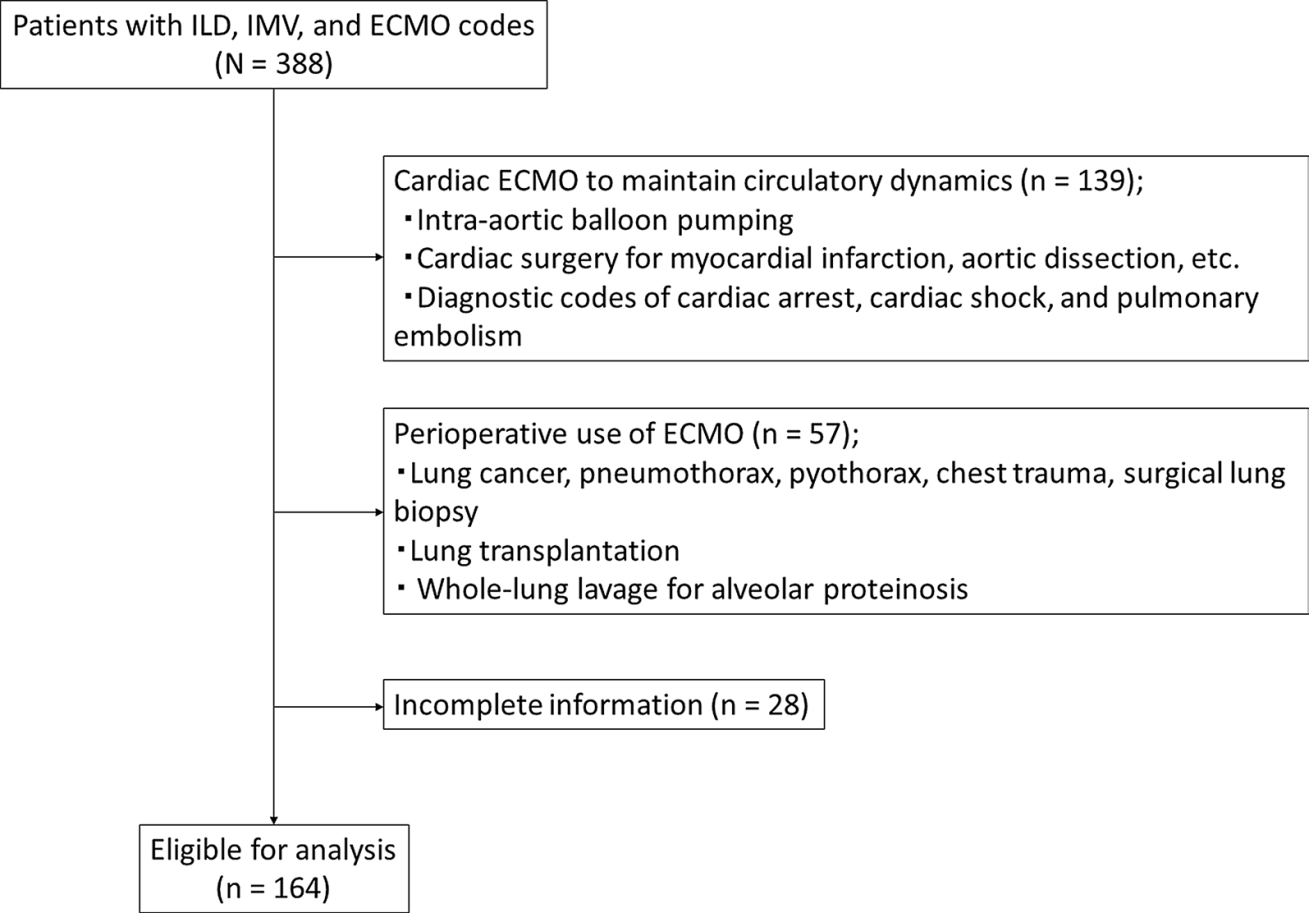
Patient selection diagram

Adult patients with both ILD codes (ICD-10: J670, J671, J672, J673, J674, J675, J676, J677, J678, J679, J700, J701, J702, J703, J704, J708, J82, J840, J841, J849, M0510, M313, M321, M330, M331, M332, M348, M351) and IMV codes were drawn from the 2010–2017 DPC database. After 388 cases with an ECMO code were found, ILD patients on ECMO for cardiac and temporary perioperative reasons were excluded to confine the study to those receiving ECMO for ARF. That is, patients with diagnostic or surgical codes pertaining to cardiovascular diseases such as coronary artery bypass grafting, intra-aortic balloon pumping, aortic dissection, cardiac arrest, and cardiogenic shock, as well as those with codes related to lung cancer surgery, lung transplantation, and pulmonary alveolar proteinosis, were excluded from this analysis (Fig. 1).

### Statistical analysis

Statistical analyses were performed using IBM SPSS statistical software (version 22; IBM SPSS, Tokyo, Japan). Continuous variables are described as medians with interquartile range (IQR), and categorical variables as frequencies and percentages. The confidence interval in two-sided analyses was set at 95%. The odds ratio of in-hospital mortality for each variable was estimated using a logistic regression model. Variables that were found to be significantly different (p < 0.05) between survivors and non-survivors on univariate analysis were entered into the multivariate analysis in a stepwise manner, with body mass index (BMI) forced into the model because of its clinical importance in recovery. Significance was defined as a p-value < 0.05 for all analyses.

## Results

This study examined 164 ILD patients undergoing ECMO for ARF in 88 hospitals, of whom 122 (74.4%) died during hospitalization. The median age was 65 years (IQR, 57–71 years), with patients over 70 years old constituting 31% of the study population. Survivors were found to be significantly younger than non-survivors; however, no significant differences were observed in sex and BMI between the two groups (Table 1).

**Table 1:** Comparison of clinical characteristics between the non-survivors group and the survivors group on univariate analysis

	Non-survivors (n = 122)	Survivors (n = 42)	Crude odds ratio	95% confidence interval
Male	83 (68.0)	30 (71.4)	1.175	0.544–2.537
Age, year	67 (58–73)	61 (53–68)	1.037	1.008–1.067
20–29	1 (0.8)	1 (2.4)	n/a	n/a
30–39	2 (1.6)	3 (7.1)		
40–49	10 (8.2)	6 (14.3)		
50–59	22 (18.0)	5 (11.9)		
60–69	43 (35.2)	20 (47.6)		
70–79	39 (32.0)	6 (14.3)		
80 ≤	5 (4.1)	1 (2.4)		
Age > 65	71 (58.2)	14 (33.3)	2.784	1.334–5.810
BMI, kg/m^2^	23.4 (21.1–26.5)	22.1 (20.1–24.6)	1.045	0.956–1.141
BMI > 30, kg/m^2^	7 (6.2)	2 (5.1)	1.222	0.243–6.145
Ambulance transportation	75 (61.5)	26 (61.9)	0.982	0.477–2.021
Hospital stay, day	30 (18–48)	48 (31–89)	0.990	0.982–0.998
Diabetes	29 (23.7)	9 (21.4)	1.143	0.490–2.666
Connective tissue disease	32 (26.2)	7 (16.7)	1.778	0.718–4.400

Data are expressed as number of patients (%) and median (IQR)

*BMI* body mass index, *IQR* interquartile range, *n/a* not assessed

As shown in Table 2, most of the patients received broad-spectrum antibiotics, high-dose systemic steroids defined as the equivalent of methylprednisolone ≥ 500 mg/day, and low-dose systemic steroids defined as the equivalent of methylprednisolone < 500 mg/day.

**Table 2:** Comparison of drugs and procedures between non-survivors and survivors on univariate analysis

	Non-survivors (n = 122)	Survivors (n = 42)	Crude odds ratio	95% confidence interval
Beta-lactam antibiotics				
Penicillin	65 (53.3)	21 (50.0)	1.140	0.565–2.300
Cephem	60 (49.2)	21 (50.0)	0.968	0.480–1.951
Carbapenem	99 (81.1)	30 (71.4)	1.722	0.767–3.865
Macrolide	41 (33.6)	25 (59.5)	0.344	0.167–0.708
Fluoroquinolone	68 (55.7)	21 (50.0)	1.259	0.624–2.542
Tetracycline	18 (14.8)	5 (11.9)	1.281	0.444–3.695
Trimethoprim-sulfamethoxazole	78 (63.9)	31 (73.8)	0.629	0.288–1.373
Anti-MRSA drugs	83 (68.0)	24 (57.1)	1.596	0.777–3.279
Anti- influenza drugs	10 (8.2)	9 (21.4)	0.327	0.123–0.873
Anti-cytomegalovirus drugs	29 (23.8)	8 (19.0)	1.325	0.552–3.182
Anti-fungal drugs	64 (52.5)	13 (31.0)	2.462	1.169–5.182
Low-dose Steroid	116 (95.1)	41 (97.6)	0.472	0.055–4.035
High-dose Steroid	105 (86.1)	33 (78.6)	1.684	0.686–4.133
High-dose cyclophosphamide	38 (31.1)	6 (14.3)	2.714	1.055–6.986
Other immunosuppressant	42 (34.4)	8 (19.0)	2.231	0.948–5.251
Protease inhibitor	66 (54.1)	14 (33.3)	2.357	1.132–4.910
Recombinant human soluble thrombomodulin	43 (35.2)	12 (28.6)	1.361	0.633–2.926
Sivelestat sodium	64 (52.5)	18 (42.9)	1.471	0.726–2.983
Duration of ECMO, day	14 (7.8–27.3)	7.5 (4.0–14.3)	1.043	1.010–1.077
Duration of ventilation, day	20.0 (14.0–36.5)	13.5 (6.8–32.8)	1.012	0.991–1.033
CHDF	79 (64.8)	16 (38.1)	2.985	1.446–6.165

Data are expressed as number of patients (%)

*CHDF* Continuous hemodialysis filtration, *ECMO* extracorporeal membrane oxygenation, *MRSA* methicillin-resistant *Staphylococcus aureus*

Patients who were treated with macrolides were concurrently administered other antibiotics in 65 of 66 cases: azithromycin in 57/66 patients (86.4%); erythromycin in 7/66 (10.6%); and clarithromycin in 6/66 (9.1%) (overlap permitted).

**Fig. 2:**
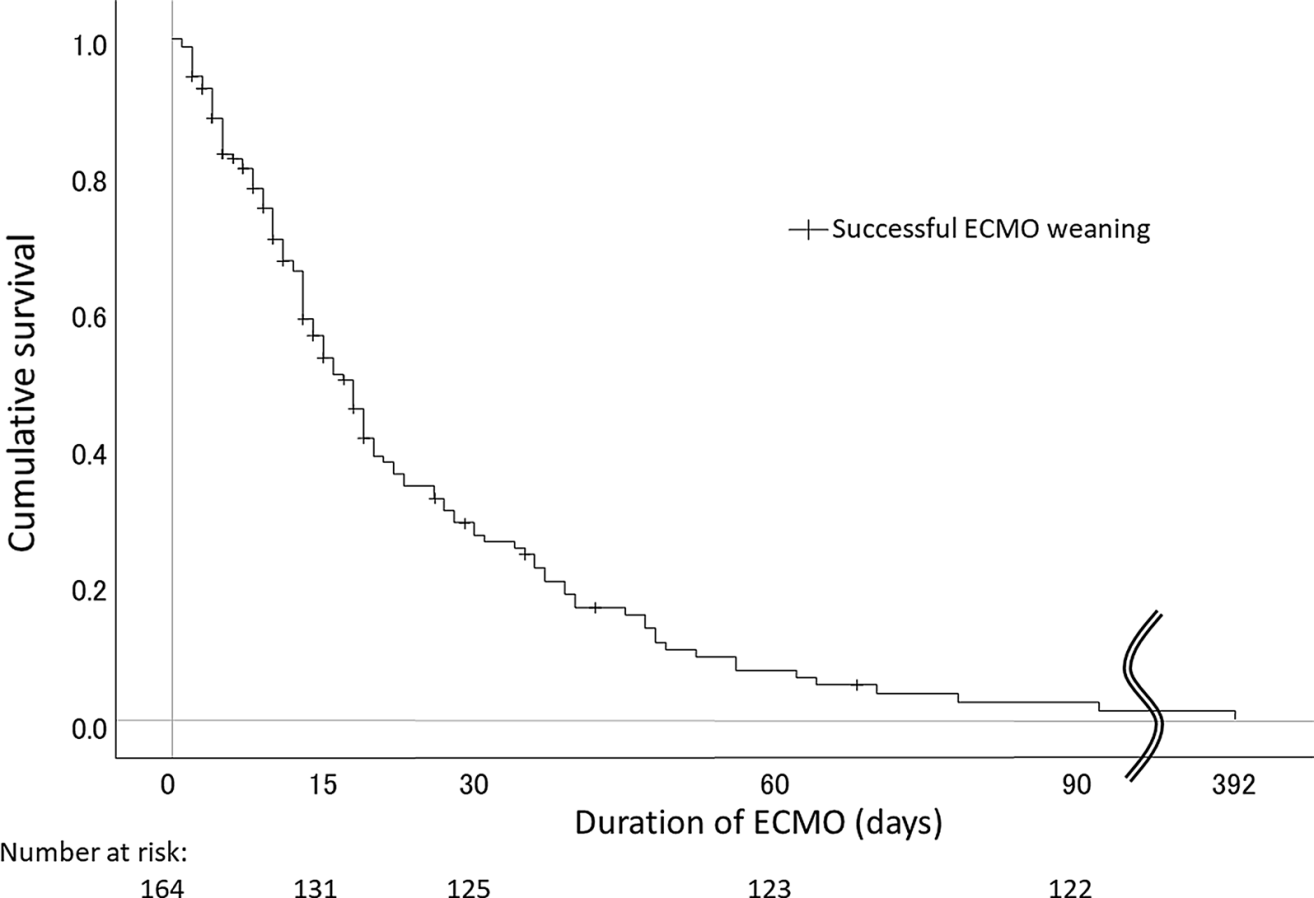
Kaplan–Meier curve plotted for cumulative survival in relation to the duration of extracorporeal membrane oxygenation for acute respiratory failure among interstitial lung disease patients

Survivors were treated more frequently with macrolides and anti-influenza drugs and less frequently with anti-fungal drugs, high-dose cyclophosphamide, and protease inhibitors. ECMO duration was significantly longer in non-survivors than in survivors, whereas duration of intubation was not significantly different between the two groups. Kaplan–Meier cumulative survival curve analysis showed that successful weaning from ECMO occurred mostly during the early days after its initiation (Fig. 2).

Multivariate analysis, which involved constructing multiple models adjusted for significant variables on univariate analysis and BMI in a stepwise manner, showed that advanced age, absence of macrolide use, and use of antifungal drugs were associated with significantly higher in-hospital mortality (Table 3).

**Table 3:** Multivariate logistic regression analysis used to identify variables associated with in-hospital death

	Adjusted odds ratio	95% confidence interval
Age, years	1.043	1.009–1.078
Macrolides	0.305	0.134–0.698
High-dose cyclophosphamide	2.530	0.912–7.017
Anti-fungal drugs	2.416	1.025–5.696
Protease inhibitor	2.218	0.945–5.209

## Discussion

This study showed that in-hospital mortality of ILD patients receiving ECMO for ARF was approximately 75%. It also demonstrated that advanced age, absence of macrolide use, and use of antifungal drugs were associated with significantly higher in-hospital mortality among these patients.

A systematic review of ILD patients treated in intensive care units without ECMO showed that mortality was 65% in patients with idiopathic pulmonary fibrosis during the period 2005–2017 and 48% in mixed ILD patients between 2010 and 2017 [2]. In the present study, the rate of in-hospital mortality (74.4%) in mixed ILD patients treated with ECMO was higher than previously reported mortality rates among patients receiving conventional treatments without ECMO. A possible reason for the higher mortality in ECMO cases is that patients treated with ECMO were generally refractory to conventional IMV, indicating the greater severity of their condition compared to the patients treated without ECMO.

The decision regarding the time of ECMO weaning in successful cases or ECMO withdrawal in refractory cases needs to be discussed carefully. In the present study, survivors were successfully weaned from ECMO after a median period of 8 days (IQR, 4–14 days) whereas ECMO was withdrawn in non-survivors at a median of 14 days (IQR, 8–27 days). Indeed, Kaplan–Meier survival curve analysis showed that successful weaning from ECMO was more frequent in the early days after its initiation. On the other hand, 67.2% of non-survivors died on the day of withdrawal, which implies that they relied entirely on ECMO as a life-sustaining procedure at the end of their lives. The present results suggest that continuation of ECMO over 14 days is less likely to produce a good outcome.

Survivors had a shorter, but not significantly, duration of ventilation than non-survivors. This variable is subject to lead-time bias. The DPC dataset was obtained from a single hospitalization at each hospital. Bias may be introduced, for example, if a previously ventilated patient was transferred to another hospital to receive ECMO therapy. The start of ventilation is considered to begin on the date of admission at the receiving hospital. In such a case, duration of ventilation would be underestimated by an unknown length of time. It is unknown how this bias may have affected the statistical comparison.

In the present study, advanced age was significantly associated with high in-hospital mortality. The Respiratory EMO Survival Prediction score [11] and Predicting Death for Severe ARDS on Veno-venous ECMO (PRESERVE) score [12] include young age as an indication for ECMO, regardless of the type of respiratory failure. In addition, the Extracorporeal Life Support Organization Guidelines for Adult Respiratory Failure published in 2017 have suggested a higher risk of a poor prognosis with increasing age [13]. Furthermore, the Extracorporeal Life Support Organization Coronavirus Disease 2019 Interim Guidelines have referred to age ≥ 65 years as contraindication for ECMO [14]. The present results in ILD patients are consistent with these guidelines. ECMO therapy consumes a considerable amount of medical resources, so the cost–benefit balance needs to be considered on a case-by-case basis, especially in elderly patients.

There was no significant difference in BMI between survivors and non-survivors, which contradicts the PRESERVE scoring system stating that a BMI value > 30 kg/m^2^ is related to a good prognosis [12]. This disparity in results could be attributed to the low number of obese patients in the present study (n = 9), which may have prevented meaningful statistical analyses. Further research is needed to determine how obesity affects the prognosis of ILD patients undergoing ECMO.

Use of macrolides was found to correlate significantly with a good prognosis in the present study. Macrolides have immunomodulatory effects [15–17], and their combined administration is capable of diminishing mortality in critically ill patients with community-acquired pneumonia [18]. In fact, an official clinical practice guideline of the American Thoracic Society and Infectious Diseases Society of America recommended macrolide-containing regimens for the treatment of hospitalized patients with severe pneumonia [19]. Furthermore, an investigation of the effectiveness of azithromycin for the treatment of acute exacerbations of idiopathic pulmonary fibrosis reported that mortality was significantly lower in patients treated with azithromycin than in those treated with fluoroquinolones [20]. Therefore, antibiotic therapy with macrolide-containing regimens might prove effective not only in patients with severe community-acquired pneumonia, but also in ILD patients receiving ECMO.

The present study also demonstrated an association between use of antifungal drugs and increased in-hospital mortality. Since observational studies are unable to determine causal relationships, whether antifungal drug use or fungal infections could have impacted the results remains unclear. Antifungal drugs are generally administered when patients are thought to develop fungal infections during immunosuppressive therapy. Thus, a diagnosis of fungal infections, rather than antifungal drug use per se, might affect a patient’s prognosis.

Use of anti-influenza virus drugs was significantly associated with survival to discharge on univariate analysis in the present study, but it was not included in the multivariate analysis. The efficacy of ECMO in H1N1-related ARDS has been reported [5]. In this regard, whereas ECMO might be beneficial for influenza-related respiratory disorder itself, its efficacy could be limited when this disease is accompanied by ILD.

The strength of this study is that a large number of patients from a nationwide database were included in the analysis. However, some limitations are derived from the retrospective nature of the study. First, the subtype of ILD was uncertain because its input was not required in the DPC database. However, antifibrotic agents covered by Japanese health insurance only for patients with idiopathic pulmonary fibrosis were used in just 12 cases during the study period. Presumably, the number of idiopathic pulmonary fibrosis cases would be limited. Second, no standardized ECMO initiation and management protocol exists among hospitals, giving rise to selection and intervention biases. Third, the level of mechanical ventilation could not be reported because the DPC database does not include this information. Finally, long-term outcomes could not be clarified owing to the lack of post-discharge information.

## Conclusions

In-hospital mortality of ILD patients receiving ECMO for ARF was found to be nearly 75%. The indications for ECMO in ILD patients who are not lung transplantation candidates should be carefully considered. Advanced age, non-use of macrolides, and use of anti-fungal drugs may be associated with a poor prognosis in ILD patients undergoing ECMO therapy.

## Data Availability

The datasets used and analyzed during the current study are available from the corresponding author on reasonable request.

## Abbreviations

ARF: Acute respiratory failure
BMI: Body mass index
DPC: The Japanese Diagnosis Procedure Combination
ECMO: Extracorporeal membrane oxygenation
ILD: Interstitial lung disease
IMV: Invasive mechanical ventilation
IQR: Interquartile range

## References

[1] Gaudry S, Vincent F, Rabbat A, Nunes H, Crestani B, Naccache JM, Wolff M, Thabut G, Valeyre D, Cohen Y, Mal H (2014). Invasive mechanical ventilation in patients with fibrosing interstitial pneumonia. J Thorac Cardiovasc Surg.

[2] Huapaya JA, Wilfong EM, Harden CT, Brower RG, Danoff SK (2018). Risk factors for mortality and mortality rates in interstitial lung disease patients in the intensive care unit. Eur Respir Rev.

[3] Gannon WD, Lederer DJ, Biscotti M, Javaid A, Patel NM, Brodie D, Bacchetta M, Baldwin MR (2018). Outcomes and mortality prediction model of critically ill adults with acute respiratory failure and interstitial lung disease. Chest.

[4] Abrams D, Brodie D (2017). Extracorporeal membrane oxygenation for adult respiratory failure: 2017 update. Chest.

[5] Davies A, Jones D, Bailey M, Beca J, Bellomo R, Blackwell N, Forrest P, Gattas D, Granger E, Herkes R (2009). Extracorporeal membrane oxygenation for 2009 influenza A(H1N1) acute respiratory distress syndrome. JAMA.

[6] Peek GJ, Mugford M, Tiruvoipati R, Wilson A, Allen E, Thalanany MM, Hibbert CL, Truesdale A, Clemens F, Cooper N (2009). Efficacy and economic assessment of conventional ventilatory support versus extracorporeal membrane oxygenation for severe adult respiratory failure (CESAR): a multicentre randomised controlled trial. Lancet.

[7] Faverio P, De Giacomi F, Sardella L, Fiorentino G, Carone M, Salerno F, Ora J, Rogliani P, Pellegrino G, Sferrazza Papa GF (2018). Management of acute respiratory failure in interstitial lung diseases: overview and clinical insights. BMC Pulm Med.

[8] Trudzinski FC, Kaestner F, Schäfers HJ, Fähndrich S, Seiler F, Böhmer P, Linn O, Kaiser R, Haake H, Langer F (2016). Outcome of patients with interstitial lung disease treated with extracorporeal membrane oxygenation for acute respiratory failure. Am J Respir Crit Care Med.

[9] Hayashida K, Murakami G, Matsuda S, Fushimi K (2020). History and profile of Diagnosis Procedure Combination (DPC): development of real data collection system for acute inpatient care in Japan. J Epidemiol.

[10] Yamana H, Moriwaki M, Horiguchi H, Kodan M, Fushimi K, Yasunaga H (2017). Validity of diagnoses, procedures, and laboratory data in Japanese administrative data. J Epidemiol.

[11] Schmidt M, Bailey M, Sheldrake J, Hodgson C, Aubron C, Rycus PT, Scheinkestel C, Cooper DJ, Brodie D, Pellegrino V (2014). Predicting survival after extracorporeal membrane oxygenation for severe acute respiratory failure. The Respiratory Extracorporeal Membrane Oxygenation Survival Prediction (RESP) score. Am J Respir Crit Care Med.

[12] Schmidt M, Zogheib E, Rozé H, Repesse X, Lebreton G, Luyt CE, Trouillet JL, Bréchot N, Nieszkowska A, Dupont H (2013). The PRESERVE mortality risk score and analysis of long-term outcomes after extracorporeal membrane oxygenation for severe acute respiratory distress syndrome. Intensive Care Med.

[13] Extracorporeal Life Support Organization (ELSO) Guidelines for Adult Respiratory Failure August, 2017. http://www.elso.org.

[14] Shekar K, Badulak J, Peek G, Boeken U, Dalton HJ, Arora L, Zakhary B, Ramanathan K, Starr J, Akkanti B (2020). Extracorporeal life support organization coronavirus disease 2019 interim guidelines: a consensus document from an international group of interdisciplinary extracorporeal membrane oxygenation providers. Asaio J.

[15] Zimmermann P, Ziesenitz VC, Curtis N, Ritz N (2018). The immunomodulatory effects of macrolides-a systematic review of the underlying mechanisms. Front Immunol.

[16] Friedlander AL, Albert RK (2010). Chronic macrolide therapy in inflammatory airways diseases. Chest.

[17] Kanoh S, Rubin BK (2010). Mechanisms of action and clinical application of macrolides as immunomodulatory medications. Clin Microbiol Rev.

[18] Sligl WI, Asadi L, Eurich DT, Tjosvold L, Marrie TJ, Majumdar SR (2014). Macrolides and mortality in critically ill patients with community-acquired pneumonia: a systematic review and meta-analysis. Crit Care Med.

[19] Metlay JP, Waterer GW, Long AC, Anzueto A, Brozek J, Crothers K, Cooley LA, Dean NC, Fine MJ, Flanders SA (2019). Diagnosis and treatment of adults with community-acquired pneumonia. An Official Clinical Practice Guideline of the American Thoracic Society and Infectious Diseases Society of America. Am J Respir Crit Care Med.

[20] Kawamura K, Ichikado K, Yasuda Y, Anan K, Suga M (2017). Azithromycin for idiopathic acute exacerbation of idiopathic pulmonary fibrosis: a retrospective single-center study. BMC Pulm Med.

